# Complete Genome Sequence of the Drug-Naive Classical *Staphylococcus aureus* Strain FDA209P

**DOI:** 10.1128/genomeA.01343-15

**Published:** 2015-11-12

**Authors:** Madhuri Singh, Takashi Sasaki, Miki Matsuo, Yuh Morimoto, Yoshifumi Aiba, Keiichi Hiramatsu

**Affiliations:** Department of Microbiology, Faculty of Medicine, Juntendo University, Tokyo, Japan

## Abstract

We report the complete genome sequence of the methicillin-sensitive *Staphylococcus aureus* (MSSA) strain FDA209P (ATCC 6538P and NCTC 7447).

## GENOME ANNOUNCEMENT

FDA209P is one of the standard strains for the species *Staphylococcus aureus* (1) and has been used in various research fields. It was isolated from a human source prior to the introduction of antibiotics, and thus this strain has never been exposed to man-made antibiotics and challenging environmental conditions. Therefore, it would be appropriate to call the FDA209P a drug-naive classical *S. aureus* strain. FDA209P belongs to the sequence type (ST) 464, and is negative for *mecA*, β-lactamase, and many other antibiotic resistance genes. However, it is positive for the tetracycline resistance gene *tet*38. It is susceptible to almost all the clinically available antibiotics, for which it is widely employed as a reference methicillin-sensitive *S. aureus* (MSSA) strain of antibiotic susceptibility assays. The complete genome sequence of this drug-naive strain will help us understand the genomic evolution and antibiotic adaptations of *S. aureus*.

The genome of strain FDA209P was sequenced using a PacBio RS II sequencer (10-kbp insert library; average read length, 9,159 bp; 88,343 reads; average depth 207) and an Illumina MiSeq platform (250-mer paired-end; 3,138,333 × 2 reads). *De novo* assembly using HGAP3 (PacBio DevNet; Pacific Biosciences) produced two circular contigs composed of a chromosome and a plasmid. In order to correct sequencing errors, we mapped the Illumina reads to the PacBio contigs and built consensus sequences using bwa-0.7.5a and samtools-0.1.19 (2, 3). Gene annotation was performed on the Rapid Annotations using Subsystems Technology (RAST) server (4) and the KEGG Automatic Annotation Server (KAAS) (5).

The FDA209P chromosomal and plasmid genomes are 2,775,733 bp (GC content, 32.9%) and 27,490 bp (GC content, 30.7%) in size, respectively. The chromosomal genome possesses 2,571 open reading frames, 6 ribosomal operons, and 57 tRNAs. There are 31 protein-coding sequences on the plasmid.

This entry of GenBank on the strain of *S. aureus* FDA209P will contribute to various research fields, such as physiology, evolution, epidemiology, and antimicrobial resistance and tolerance in *S. aureus*.

### Nucleotide sequence accession numbers.

The complete genome sequence of *S. aureus* strain FDA209P has been deposited in GenBank under the accession numbers AP014942 and AP014943 for the chromosome and plasmid, respectively.
